# Impact of SMS Text Messaging Reminders on Helmet Use Among Motorcycle Drivers in Dar es Salaam, Tanzania: Randomized Controlled Trial

**DOI:** 10.2196/27387

**Published:** 2022-04-07

**Authors:** Benjamin Campbell, Jesse Heitner, Peter Amos Mwelelo, Alexis Fogel, Vaidehi Mujumdar, Lisa V Adams, Respicious Boniface, Yanfang Su

**Affiliations:** 1 School of Education and Social Work University of Sydney Sydney Australia; 2 International Clinical Research Center Department of Global Health University of Washington Seattle, WA United States; 3 Petmos Attorneys Dar es Salaam United Republic of Tanzania; 4 Benioff Children’s Hospital Oakland University of California San Francisco San Francisco, CA United States; 5 Department of Obstetrics and Gynecology Ichan School of Medicine Mount Sinai New York City, NY United States; 6 Department of Obstetrics and Gynecology New York City Health and Hospitals Queens New York City, NY United States; 7 Section of Infectious Disease and International Health Department of Medicine Geisel School of Medicine at Dartmouth Hanover, NH United States; 8 Anaesthesiology Department School of Medicine Muhimbili University of Health and Allied Sciences Dar es Salaam United Republic of Tanzania; 9 Department of Global Health University of Washington Seattle, WA United States

**Keywords:** road traffic injury, behavior change, SMS reminders, mobile health, vehicle safety, mHealth, SMS, traffic injuries, transportation, public transportation, safety, automotive, automotive safety

## Abstract

**Background:**

Road traffic injury is a pressing public health issue in Tanzania. Increasing helmet use among motorcycle drivers can help reduce the burden due to road traffic injuries in the country. Helmet adherence can be supported through mobile health interventions.

**Objective:**

The aim of this study is to evaluate the comparative impact of two different types of SMS text messaging reminders on motorcycle helmet use.

**Methods:**

Participants were 391 commercial motorcycle taxi drivers in Dar es Salaam, Tanzania. Participants were randomized into three groups, each receiving a different set of messages: (1) social norming messages aimed at emphasizing society’s positive stance on helmet wearing, (2) fear appeal messages that emphasized the dangers of riding without a helmet, and (3) control group messages, which included basic road safety messages unrelated to helmet use. Every participant received the control messages. Adherence to helmet use was evaluated by self-report through surveys conducted at baseline, 3 weeks, and 6 weeks.

**Results:**

At 6 weeks, the odds of self-reporting consistent helmet use were estimated to be 1.58 times higher in the social norming group than in the control group (*P*=.04), though this difference was not significant after accounting for multiple testing. There was little difference between fear appeal and control group recipients (odds ratio 1.03, *P*=.47). Subgroup analysis suggests that both fear appeal and social norming message types might have been associated with increased helmet use among participants who did not consistently wear helmets at baseline (odds ratio 1.66 and odds ratio 1.84, respectively), but this was not significant (*P*=.11 and *P*=.07, respectively). Among those who were consistent wearers at baseline, the social norming messages performed better than the fear appeal messages, and this difference reached traditional significance (*P*=.03), but was not significant after accounting for multiple testing.

**Conclusions:**

The use of SMS text messaging reminders may improve helmet use among motorcycle drivers when framed as social norming messages. Given that nearly half of the drivers in our sample did not consistently wear their helmets on every trip, strategies to increase consistent usage could greatly benefit public safety.

**Trial Registration:**

ClinicalTrials.gov NCT02120742; https://clinicaltrials.gov/ct2/show/NCT02120742

## Introduction

Road traffic injury is a pressing public health issue in Tanzania. According to the Global Burden of Disease 2017 Study, road traffic injury is the fourth leading cause of disability-adjusted life years for men aged 15-49 years in Tanzania [1]. Men are particularly at high risk of road traffic injury because nearly all drivers of motorcycle taxis (“bodabodas” in Kiswahili, or “bodas” for short), a major form of public transportation in the country, are men.

Studies have shown that helmet use can significantly reduce disability and death resulting from road traffic injuries [2]. Research conducted in Tanzania has shown that a lack of helmet wearing increases the probability of fatality in a motorcycle accident [3]. Efforts have been made by the Tanzanian government to develop tighter helmet use laws [4]. However, adherence to helmet use has remained dangerously low throughout the country [5,6]. This is partly because enforcement of laws is so limited [4].

One promising intervention to promote helmet wearing is the use of persuasive SMS text messaging reminders delivered to boda drivers. There is substantial evidence that mobile health interventions using SMS text messaging can lead to behavior change. For instance, in the largest study of its kind, texts reminding participants not to smoke significantly increased the likelihood that someone in a smoking cessation program would stop smoking [7]. Other studies have shown that text reminders can dramatically improve adherence to medication regimens [8,9]. Because of the high prevalence of cell phone and SMS text messaging use in Tanzania, especially among young people, the context is appropriate for such an intervention [10]. We implemented an innovative program that delivers SMS text messages to boda drivers over a 6-week period, reminding and persuading them to wear their helmets. To date, no program like this has been rigorously evaluated via randomized trial. The literature suggests that it takes approximately 21-42 days to form a new habit, so a 6-week study period was determined to be a sufficient time period to measure changes in helmet use [11,12]. The rate of consistent helmet wearing at the study’s 6-week endpoint is the primary outcome of interest.

A key goal of this study is to measure which type of message leads to the greatest increase in helmet use. Messaging based on the social norming model, which promotes behavior change by informing the target population of how most people behave [13], could potentially be a more effective way of communicating road safety messages, particularly for men, compared to appealing to a fear of negative health outcomes, which is a longstanding health communication method. Findings from two recent studies support this idea by showing that road safety advertisements with threats of social consequences, such as the threat of losing one’s driving license, were more effective at changing young males’ driving behaviors than were advertisements depicting harsh physical consequences [14,15]. The fear appeal method, while historically prominent in the field of public health, has more recently been shown to be ineffective in leading to behavior change, especially among young men [16]. For example, a study by Woolley et al [16] demonstrated that men often dissociate their own speeding behaviors from a social problem and therefore perceive related fear appeals as being directed more toward others than themselves. This is consistent with a broader trend in the psychology of aging literature wherein younger adults are more motivated by potential rewards than loss aversion, a balance that reverses in older adulthood [17]. In this study, we aimed to test social norming and fear appeal messages against a control and against each other to see which, if either, has a greater impact on helmet use. Our trial implementation was successful and our results tentatively favor one type of message over the other.

## Methods

### Study Design

We conducted a randomized controlled trial to evaluate the impact of an SMS text messaging program on helmet use among boda drivers in Dar es Salaam, Tanzania. Participants were recruited in a convenience sample from the population of boda drivers in 3 districts in Dar es Salaam. Boda drivers were approached at boda stands with 3 or more boda drivers waiting for clients. The inclusion criteria required participants to be ≥18 years old, own a mobile phone with SMS text messaging capabilities, demonstrate the ability to retrieve SMS text messages, and have access to a helmet. Preintervention power calculations indicated that 385 participants would be needed to detect a 20 percentage point increase in consistent helmet use over an anticipated baseline of 32.4% being consistent users. In total, 391 participants were recruited. There were no incentives to join the study.

All participants were informed that they would receive 3 SMS text messages per week. A prestudy questionnaire indicated that the best time to deliver SMS text messages was between 6 AM and 7 AM, during off-peak hours. Participants were randomized into one of three study arms, with each group receiving a different type of message: (1) social norming (eg, “Most of your peers properly wear their helmet every day – do you?”); (2) fear appeal (eg, “If you do not wear your helmet while driving, you will increase your chances of injury”); and (3) control, which included basic road safety messages (eg, “This is a short reminder to not speed while driving your boda”). Groups 1 and 2 received the control messages in addition to their group-specific messages. The information in the group-specific messages was designed to be both motivational and accurate, and was based on a literature review of motorcycle helmet use and road safety in Tanzania and the surrounding region. Participants received the intervention between May and June 2014. Texts were delivered in the local language, Kiswahili, using a mass-messaging platform called MightyText. Texts were sent Monday, Wednesday, and Friday mornings. For the complete message list and the literature source [2,18-23] for each message, see Multimedia Appendix 1.

Randomization proceeded in a 4-step process that created matched triplets of drivers and randomly assigned one member from each triplet to each study arm. First, a logistic regression of baseline consistent helmet use on demographic and driving-habit covariates was used to create a propensity score for predicting baseline helmet use. Second, participants were stratified into two groups: those who at baseline reported they had consistently worn their helmet on all trips in the past two weeks, and those who reported inconsistent use. Third, within the two strata, triplets were made, beginning by grouping the 3 individuals with the lowest use, then the next 3 lowest, and so on, in the same “propensity triplet.” Only one individual with the highest score remained unmatched into a triplet. Finally, for each triplet, an integer from 1 to 6 was randomly drawn with replacement. Each integer represented one of the 6 permutations by which 3 (ordered) individuals may be assigned (one each) to 3 different treatments: ABC, ACB, BAC, BCA, CAB, and CBA. The individuals in the triplet were thereby simultaneously assigned to an arm of the study, with one member in each treatment arm. The last individual with the highest score was similarly assigned, and treated as the lowest score in their own triplet.

Matching in this fashion had two aims. First, it created equally sized treatment arms, which maximized statistical power across the planned group comparisons. Second, it was intended to balance the drivers’ unobservable propensity to wear helmets across treatment arms by ensuring that baseline helmet use and predicted helmet use were balanced across treatment arms. Stratification assured that equal numbers of consistent wearers and inconsistent wearers were in each study arm, and matching into triplets based on close propensity scores prior to random assignment assured that estimated propensity to consistently wear helmets was also balanced across treatment arms. Matching on a propensity score constructed from observable covariates has been shown to be sufficient to remove bias from all covariates used to construct the propensity score [24]. Though this technique was originally conceived to improve causal inference in observational studies, matching on relevant covariates before treatment assignment in randomized experiments is now a common practice that can increase efficiency of estimation and the power of hypothesis tests [25]. Moreover, inadvertently matching on irrelevant covariates prior to a random assignment does not harm statistical efficiency or power [25].

Participant adherence to helmet use was captured through self-report surveys at baseline, at the 3-week midpoint of the experiment, and at 6 weeks (conclusion of the experiment).

### Ethics Approval

This study was approved by the Institutional Review Board (Committee for the Protection of Human Subjects) at Dartmouth College, United States (00024570), and the Ethics Review Committee at Muhimbili University of Health and Allied Sciences, Tanzania.

### Study Population Baseline Characteristics

The baseline characteristics of the study population are shown in Table 1. The mean age of participants was 28 years, all participants were men, and a majority had at most an elementary level education. At baseline, approximately 53% (207/391) of participants claimed that they wore their helmet on every trip, which was more than the 32% anticipated from previous literature review, on which our power calculations were based. There were no statistically significant differences across treatments for any observed variable. Self-reporting of consistent helmet wearing was perfectly balanced across treatment arms by the stratified design of the randomization method.

**Table 1:** Balance check for all observed baseline variables.

Baseline variable		Social norming	Fear appeal	Control	Test statistic^a^		*P* value	
**District (n=391), n (%)**						*X*^2^(4)=1.71		.79
	District 1	51 (39.2)	48 (36.6)	42 (32.3)				
	District 2	55 (42.3)	61 (46.6)	62 (47.7)				
	District 3	24 (18.5)	22 (16.8)	26 (20)				
Age, years (n=384), mean (SD)		28.6 (6.4)	27.6 (6.7)	27.9 (5.9)	*F*=0.86		.43	
**Education (n=375), n (%)**						*X*^2^(2)=0.57		.75
	Elementary or none	86 (67.7)	85 (69.1)	90 (72)				
	Junior high or above	41 (32.3)	38 (30.9)	35 (28)				
Currently married (n=381), n (%)		83 (65.4)	85 (67.5)	84 (65.6)	*X*^2^(2)=0.15		.93	
Has children (n=378), n (%)		79 (62.7)	82 (66.1)	81 (63.3)	*X*^2^(2)=0.37		.83	
Cell phone self-owned (n=380), n (%)		128 (100)	126 (100)	124 (98.4)	*X*^2^(2)=4.05		.13	
Household size (n=386), mean (SD)		5.19 (2.4)	5.28 (3.2)	4.93 (2.2)	*F*=0.59		.56	
**Primary driving setting (n=380), n (%)**						*X*^2^(4)=2.81		.59
	Urban/downtown	25 (19.7)	29 (22.3)	30 (24.4)				
	Suburban/residential	24 (18.9)	32 (24.6)	23 (18.7)				
	Both equally	78 (61.4)	69 (53.1)	70 (56.9)				
**Night driving frequency (n=389), n (%)**						*X*^2^(6)=3.00		.81
	Never	34 (26.4)	42 (32.3)	36 (27.7)				
	Sometimes	41 (31.8)	43 (33.1)	45 (34.6)				
	Usually	32 (24.8)	26 (20)	33 (25.4)				
	Always	22 (17.1)	19 (14.6)	16 (12.3)				
Wears helmet consistently (n=391), n (%)		69 (53.1)	69 (52.7)	69 (53.1)	*X*^2^(2)=0.01		>.99	
**Speeding frequency (n=390), n (%)**						*X*^2^(6)=3.55		.74
	Never	15 (11.5)	18 (13.9)	20 (15.4)				
	Sometimes	41 (31.5)	42 (32.3)	36 (27.7)				
	Usually	65 (50)	64 (49.2)	70 (58.9)				
	Always	9 (6.9)	6 (4.6)	4 (3.1)				
**Weekend driving (n=391), n (%)**						*X*^2^(6)=8.71		.19
	Never	9 (6.9)	3 (2.3)	7 (5.4)				
	Sometimes	21 (16.2)	36 (27.5)	33 (25.4)				
	Usually	30 (23.1)	33 (25.2)	32 (24.6)				
	Always	70 (53.9)	59 (45.0)	58 (44.6)				

^a^Chi-square tests were conducted for all variables except for the age and household size variables, for which an analysis of variance was conducted.

### Statistical Methods

The primary outcome of the study was self-reported adherence to helmet use as measured by the question, “In the past week, how often did you wear your helmet: (1) Every trip; (2) Not every trip.” We compared the adherence rate between experimental groups and between each experimental group and the control. A secondary outcome was heterogeneity of treatment effect by baseline helmet use habits.

A reliance on self-reports potentially introduces measurement error due to possible social desirability bias. Because helmet use is legally required, participants may have reported wearing them more frequently so as to be viewed positively. We aimed to overcome the social desirability bias by ensuring that the survey responses were anonymous. One indication that this strategy may have been successful is shown in survey respondents’ self-reported frequency of speeding; interestingly, 56% (218/390) of respondents were willing to admit to exceeding speed limits “frequently” or “always.” Another 30% (129/390) reported speeding at least “sometimes.” Speeding would be expected to be subject to the same social desirability bias as helmet use, but many respondents were willing to self-report this behavior in the anonymous survey.

To investigate the effect of treatment arm assignment, several logistic regressions of consistent helmet use on treatment assignment were run. All statistical analyses were performed using R (version 3.0.2; R Foundation for Statistical Computing). All specifications were structured to estimate an intent-to-treat effect. For all group comparisons in all specifications, statistical significance of group difference was performed by permutation analysis as follows. First, the specification was run on all data using the true treatment assignment. The analysis was then rerun with each triplet of individuals (falsely) rerandomized with replacement to one of the 6 possible permutations of treatment assignments for that triplet. Performing this analysis with many permutations wherein analyzed treatment assignments have no relation to the intervention or associated outcomes recreates the distribution of the null hypothesis in which treatment and outcomes are unrelated. The analysis was run with 5000-10,000 permutations (depending on the specification), and significance was assessed by the percentage of runs in which the null distribution yielded results of larger magnitude than that of the true treatment assignment. Permutation tests have been shown to be valid for conducting any test of a null hypothesis of no treatment effect within an experimental sample, conditional on the single requirement that treatment has been randomly assigned [26].

## Results

Our intervention was delivered over a 6-week period, with helmet use measurement at week 3 and week 6. The primary outcome of interest is the proportion of self-reported consistent helmet use at week 6. Unadjusted levels of reported helmet use for each group at both time points are shown in Table 2.

The final row represents the difference between a group’s week 6 difference from control and the group’s baseline difference from control.

Potential heterogeneity of effect by randomization strata was investigated by analyzing participants in two subgroups based on whether they were or were not consistent helmet wearers at baseline. Intervention effects had strong potential to be different in magnitude between these strata because the mechanism of effect was necessarily different between these groups. Among already consistent wearers, the only possible mechanism of effect is maintenance of adherence; conversely, for the inconsistent wearers, the only possible mechanism is promotion of adherence among the not yet adherent. Knowledge about heterogeneity or consistency of effect is important for future targeting of interventions. Unadjusted results are shown in Table 3.

The results in Table 2 show that the fear appeal and control groups had little change over the 6-week period. However, the group receiving social norming SMS text messages showed a final 11.1% lead over the control group in consistent helmet wearing despite their initial equal levels.

The results in Table 3 potentially indicate even more striking differences between treatment arms. Among drivers always wearing their helmets at baseline, the social norming arm had 9.6% more drivers stay consistent than the control arm, and the fear appeal group actually had 8.3% fewer drivers stay consistent, potentially denoting a detrimental effect of fear messages in this subgroup. Among drivers that began as inconsistent helmet wearers, 36% (18/50) of the control group became consistent helmet wearers, but the gains in the fear appeal and social norming arms were even larger (28/58, 48.3% and 29/57, 50.9%, respectively).

**Table 2:** Drivers reporting helmet use every trip (all time points).

Observations	Control	Fear appeal	Social norming
Baseline, n/N (%)	69/130 (53.1)	69/131 (52.7)	69/130 (53.1)
Difference from control, %	Reference	–0.4	0.0
Week 3, n/N (%)	62/113 (54.9)	62/117 (53)	70/122 (57.4)
Difference from control, %	Reference	–1.9	2.5
Week 6, n/N (%)	58/110 (52.7)	63/118 (53.4)	74/116 (63.8)
Difference from control	Reference	0.7	11.1
Week 6 difference in differences	Reference	1.1	11.1

**Table 3:** Drivers reporting helmet use for every trip at 6 weeks, by baseline answer.

Helmet use			Control		Fear appeal		Social norming
**Subgroup: baseline “consistent wearers”**							
	Week 6, n/N (%)	40/60 (66.7)		35/60 (58.3)		45/59 (76.3)	
	Difference from control	Reference		–8.3		9.6	
**Subgroup: baseline “inconsistent wearers”**							
	Week 6, n/N (%)	18/50 (36)		28/58 (48.3)		29/57 (50.9)	
	Difference from control	Reference		12.3		14.9	

Hypothesis testing of group-level differences was performed with logistic regression and *P* values were generated via nonparametric permutation testing to account for the correlations induced by the multistep randomization process. Regressions unadjusted for any covariates are shown in Table 4, which tests the odds ratios associated with the risk differences presented in Table 2 and Table 3. The first two columns display test results of whether each treatment arm statistically differed from the control arm. The final column of Table 4 shows tests of whether and how the effects of the two treatment arms statistically differ from each other. The first row of Table 4 presents these tests using all observations. The second and third rows present these same tests within the two subgroups of baseline “always wearers” and baseline “inconsistent wearers.” Whether there was a heterogeneous effect of treatment assignment by this baseline subgrouping is displayed in the last row of Table 4, which tests for effect modification by taking the ratio of the odds ratios between the subgroups and testing whether this ratio is significant via permutation.

In comparing the two intervention arms to the control arm, 1-sided tests of significance were used, justified by the strong a priori expectation that the two message types would only encourage, not discourage, helmet wearing. However, because we had no such a priori expectation that one messaging intervention would work better than the other, a 2-sided test was used whenever comparing the social norming and fear appeal groups.

Using all observations in an unadjusted analysis, participants in the social norming arm had odds of consistently wearing their helmet that were 1.58 times the odds of the control group, which was the strongest measured association. Jointly testing all three possible group comparisons among all participants is this study’s primary, trial-registered outcome, and it was preplanned to use a Holm-Bonferroni correction to account for this multiple testing. The 1-sided *P* value of .04 comparing the social norming arm to the control arm was not enough to satisfy the Holm-Bonferroni cutoff for simultaneously testing 3 null hypotheses, which requires that the most significant of 3 *P* values be less than or equal to .05/3 (.0167) to set a maximum Type I family-wise error rate of .05.

Within the subgroup of participants that started as consistent helmet wearers, neither intervention arm differed significantly from the control arm. The social norming group was measured to have 2.30 times the odds of the fear appeal group of consistently wearing their helmets (*P*=.03). However, this is nonsignificant under the Holm-Bonferroni correction for simultaneously testing 3 group differences in this subset. In the subgroup of participants that were not consistent users at baseline, both intervention arms outperformed the control group, but their gains, while perhaps clinically meaningful in size, were not statistically significant at a threshold of *P*<.05. Finally, the lowest section of Table 4 investigates whether the same message arms had different effects between the two subgroups (baseline “always wearers” and baseline “inconsistent wearers”). Although the measured effects had seemingly large differences across subgroups, these differences had *P* values well above .05.

After the unadjusted analysis, the same set of logistic regressions was performed including a set of demographic factors and baseline driving habits as controls. These controls were marital status, driving setting (primarily downtown or primarily suburban portions of the city), frequency of driving at night, and frequency of driving on the weekend. This list of controls was somewhat smaller than originally intended for several reasons. Originally, age and whether the driver had children were intended to be included in the controls, but strong multicollinearity between age, marital status, and having children precluded using all three simultaneously. Marital status was deemed to be the best summary indicator of the three as its effect was most consistent and interpretable across specifications. In addition, large amounts of missingness in self-reported income precluded its inclusion as a control variable. Table 5 reports the results of the adjusted logistic regressions.

The results in Table 5 follow those in Table 4 with relatively minor deviations. Given that the included variables were part of the original propensity score matching, it is unsurprising that their inclusion fails to alter the analysis in any meaningful way.

**Table 4:** Pairwise treatment group comparisons of odds of consistent helmet wearing (using coefficient results of unadjusted logistic regression).

Subgroup analysis		Fear appeal: control group comparison	Social norming: control group comparison	Social norming: fear appeal group comparison
**All observations**				
	Odds ratio	1.03	1.58	1.54
	*P* value^a^	.47^b^	.04^b^	.12^c^
**Subgroup: baseline “always wearers”**				
	Odds ratio	0.70	1.61	2.30
	*P* value^a^	.81^b^	.11^b^	.03^c^
**Subgroup: baseline “inconsistent wearers”**				
	Odds ratio	1.66	1.84	1.11
	*P* value^a^	.11^b^	.07^b^	.80^c^
**Subgroup effect modification**				
	Ratio of odds ratios	0.42	0.87	2.07
	*P* value^a^	.16^c^	.82^c^	.21^c^

^a^All *P* values determined by permutation analysis.

^b^One-sided test.

^c^Two-sided test.

**Table 5:** Pairwise treatment group comparisons of odds of consistent helmet wearing (using coefficient results of covariate-adjusted logistic regression).

Subgroup analysis			Fear appeal: control		Social norming: control		Social norming: fear appeal
**All observations**							
	Odds ratio	1.01		1.57		1.55	
	*P* value^a^	.49^b^		.06^b^		.12^c^	
**Subgroup: baseline “always wearers”**							
	Odds ratio	0.62		1.58		2.54	
	*P* value^a^	.86^b^		.15^b^		.03^c^	
**Subgroup: baseline “inconsistent wearers”**							
	Odds ratio	1.84		1.90		1.03	
	*P* value^a^	.09^b^		.08^b^		.93^c^	
**Subgroup effect modification**							
	Ratio of odds ratios	0.34		0.83		2.46	
	*P* value^a^	.07		.76^c^		.15^c^	

^a^All *P* values determined by permutation analysis.

^b^One-sided test.

^c^Two-sided test.

## Discussion

### Principal Findings

The results of our study show that social norming messages are potentially effective at increasing helmet use among motorcycle taxi “boda” drivers in Dar es Salaam, Tanzania. Over the 6-week period, the group receiving social norming SMS text messages showed an increase in helmet use from 53.1% to 63.8%, and that increase achieved traditional significance (*P*<.05) when compared to the control group (*P*=.04). However, accounting for multiple testing, we cannot reject the null of no association, as this *P* value is above the required *P≤*.0167 to maintain a family-wise Type I error rate of at most .05 when making 3 group comparisons. In contrast, the fear appeal and control groups showed little change over the 6-week period.

Although the main finding shows that the group receiving social norming messages increased helmet adherence the most, though not to a statistically significant degree, the findings also suggest that responsiveness to messages may also have been determined by participant baseline response. Specifically, for those who reported not wearing helmets all the time at baseline, both social norming and fear appeal messages were associated with higher adherence after the 6-week study period compared to the control group. Though shy of statistical significance due to the power limitation of restricting the sample, the associated odds ratios imply a near doubling of the odds of consistent usage, and the close similarity of the odds ratios between the two treatment arms suggests that initially inconsistent wearers are equally sensitive to both types of messages. However, among those who reported consistent helmet wearing at baseline, those recipients of social norming messages maintained high levels of adherence, while those receiving fear appeal messages actually decreased their level of consistent wearing compared to the control. Although neither treatment is associated with a statistical difference from the control in this subgroup, the combination of a positive association in the social norming arm and a deleterious association in the fear appeal arm results in a traditionally significant improvement of the social norming arm over the fear appeal arm (odds ratio 2.30, *P*=.03). However, this association does not meet the Holm-Bonferroni requirement of *P≤*.0167.

These findings have important potential implications for policy makers as well as other stakeholders in road safety. Because social norming messaging overall showed a potentially greater association with consistent helmet use than fear appeal messaging, it could be strategic for regulators and nongovernmental organizations focusing on road traffic safety to use social norming messages for any mass message or media campaigns to promote road safety and behavior change among drivers. However, a larger and more highly powered study would be required to confirm this differential association. Finally, intervention designers should note that behavior change may take some time to set in among drivers; group-level differences were noticeable at 6 weeks, but not after 3 weeks.

### Limitations

There are several limitations to this study. First, self-reports introduce the possibility of social desirability bias among the respondents thanks to the legal requirement that helmets be worn at all times. A second potential bias in this study is simply recall bias. Our main outcome question asks for an estimate of helmet use in the past week of boda driving. It is possible that drivers had difficulty remembering with accuracy the level of helmet wearing during that time. However, we believed that asking about behavior over the past week was a reasonable amount of time to ensure accuracy of estimates. Moreover, the recall burden is much lower when recalling consistency than when recalling the number of times something occurred or other numeric answers. Third, while the results can be useful in a Tanzanian urban context, they may not be applicable to other contexts. Fourth, the study was conducted in a convenience sample. The representativeness of the sample for Dar es Salaam boda drivers is left unknown. Fifth, our study measures effects of the intervention right after completion of the 6-week trial. How long measured effects persist into the future is unknown. Finally, our study is focused on helmet usage, while the ultimate goal of such an intervention is better health and safety for drivers on the road. This study was not structured or powered to detect differences in health outcomes by treatment arm, and further research would be necessary to determine if such a messaging intervention would improve health outcomes for drivers.

### Conclusions

Though the evidence is not fully conclusive, this study suggests that SMS text messaging reminders can be an effective way to improve helmet use among motorcycle drivers. Specifically, social norming messages appear to be more effective than fear appeal messages when trying to increase helmet use among boda drivers. Furthermore, for drivers who already wear their helmet consistently, fear appeal messages may actually have a detrimental effect on helmet use. Future research should further investigate whether social norming messages are more effective than fear appeals when trying to change behavior.

## Appendix

Multimedia Appendix 1 Full-text message bank.

Multimedia Appendix 2 CONSORT-eHEALTH checklist (V 1.6.1).
