# Supplementary material for: Impact of SMS Text Messaging Reminders on Helmet Use Among Motorcycle Drivers in Dar es Salaam, Tanzania: Randomized Controlled Trial
Source: J Med Internet Res. 2022 Apr 7;24(4):e27387. doi: 10.2196/27387 (PMC9030911; doi:10.2196/27387)
Supplement: Multimedia Appendix 1 [file jmir_v24i4e27387_app1.docx]

Multimedia Appendix 1. Full-text message bank.

**Social Norming**

| English | Most boda drivers in Dar wear their helmet every day they drive on the street. |
| --- | --- |
| Swahili | Madereva wengi wa bodaboda wa Dar wanavaa helmet kila siku wanapoendesha mitaani. |

Source: Amend observational study

| English | Did you know that most boda drivers on [X road]* wear their helmet every day? |
| --- | --- |
| Swahili | Je unafahamu kwamba madereva wengi wa bodaboda kwenye barabara (X) huvaa helmet kila siku? |

Source: Amend observational study

* Majority of observed boda drivers on Pugu road, New Bagamoyou Road, Morogoro Road were wearing helmet

| English | Most of your peers properly wear their helmet every day – do you? |
| --- | --- |
| Swahili | Wenzako wengi huvaa kwa usahihi helmet zao kila waendeshapo bodaboda- na wewe je? |

Source: Amend observational study

| English | Most boda drivers believe wearing their helmet is important even for short trips |
| --- | --- |
| Swahili | Madereva wengi wa bodaboda wanaamini kuvaa helmeti ni muhimu hata kwa safari fupi |

Source: Mwakapasa 2011

| English | Most boda drivers believe that wearing a helmet is important even during hot weather |
| --- | --- |
| Swahili | Madereva wengi wa bodaboda wanaamini kuvaa helmeti ni muhimu hata wakati wa joto |

Source: Mwakapasa 2011

| English | Most boda drivers in Dar say that wearing their helmet regularly is easy and comfortable |
| --- | --- |
| Swahili | Madereva wengi wa bodaboda wanasema kuvaa helmeti mara kwa mara ni rahisi na vizuri |

Source: Mwakapasa 2011

**Fear Appeal**

| English | Helmets decrease the chance of you dying in an accident. |
| --- | --- |
| Swahili | Helmet inapunguza nafasi ya wewe kufa kwenye ajali. |

Sources [18], [19], [2], [20], [21], [22], [23]

| English | Road traffic accidents are the number 1 cause of death for boda drivers in Tanzania. Make sure to wear your helmet. |
| --- | --- |
| Swahili | Ajali za barabarai ni sababu namba 1 ya vifo kwa madereva wa bodaboda Tanzania. Hakikisha unavaa helmet yako. |

Source: Global Burden of Disease 2010 Study

| English | If you do not wear your helmet while driving, you will increase your chances of injury. |
| --- | --- |
| Swahili | Ikiwa hauta vaa helmet yako wakati unaendesha, utaongeza nafasi ya kuumia. |

| English | Boda boda’s are a very risky form of transportation. Make sure to wear your helmet to prevent injury. |
| --- | --- |
| Swahili | Usafiri wa bodaboda ni hatari sana. Hakikisha unavaa helmeti kuzuia hatari. |

| English | The number of boda accidents increases every year. Make sure to wear your helmet. |
| --- | --- |
| Swahili | Idadi ya ajali za bodaboda zinaongezeka kila mwaka. Hakikisha unavaa helmeti yako. |

Source: Amend study

| English | You are more likely to have serious head injuries if you get in an accident without a helmet. |
| --- | --- |
| Swahili | Unauwezekano mkubwa wa kupata majeraha ya kichwa wakati wa ajali kama usipokuwa na helmeti. |

**Control**

| English | This is a short reminder to not speed while driving your boda. |
| --- | --- |
| Swahili | Hii ni kuku-kumbusha kuwa usiendeshe bodaboda yako kwa mwendo kasi. |

| English | This is a short reminder to follow traffic signs while driving your boda. |
| --- | --- |
| Swahili | Hii ni kuku-kumbusha kufuata alama za barabarani wakati unaendesha bodaboda yako. |

| English | This is a short reminder to make sure your passengers are safe on your boda boda. |
| --- | --- |
| Swahili | Hii ni kuku-kumbusha kuhakikisha kuwa abiria wako wapo salama kwenye bodaboda yako. |
